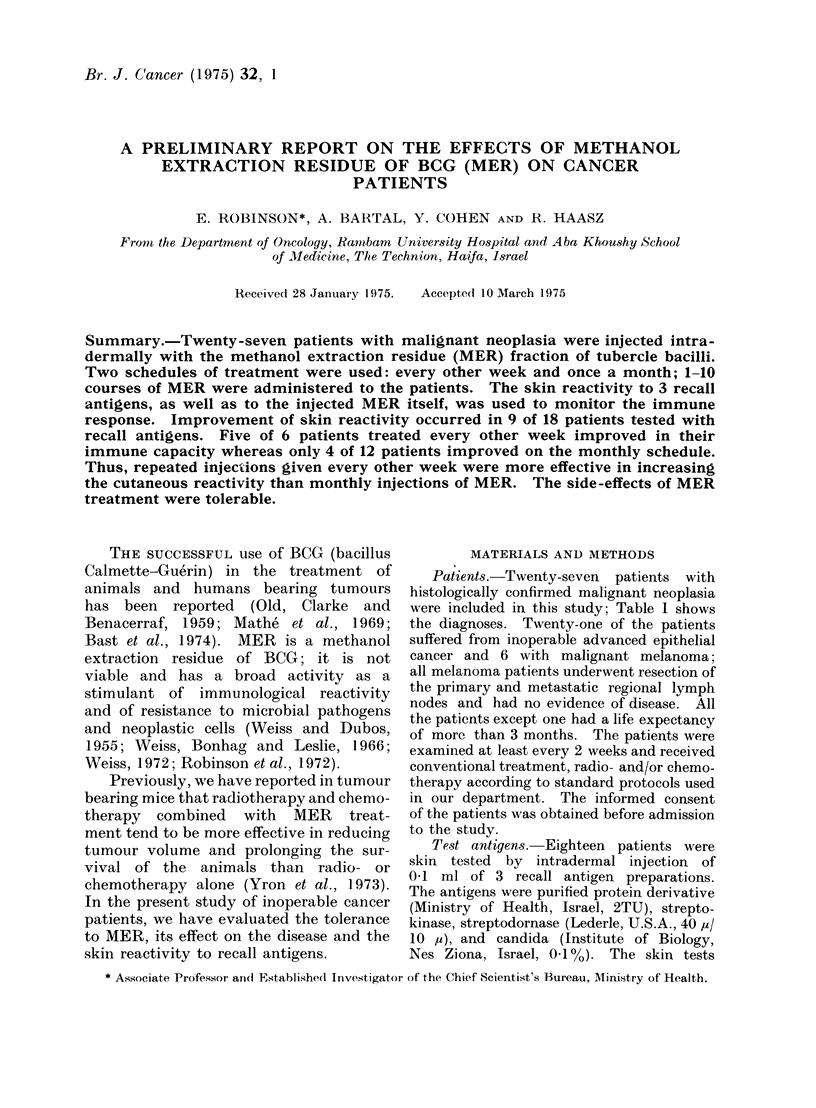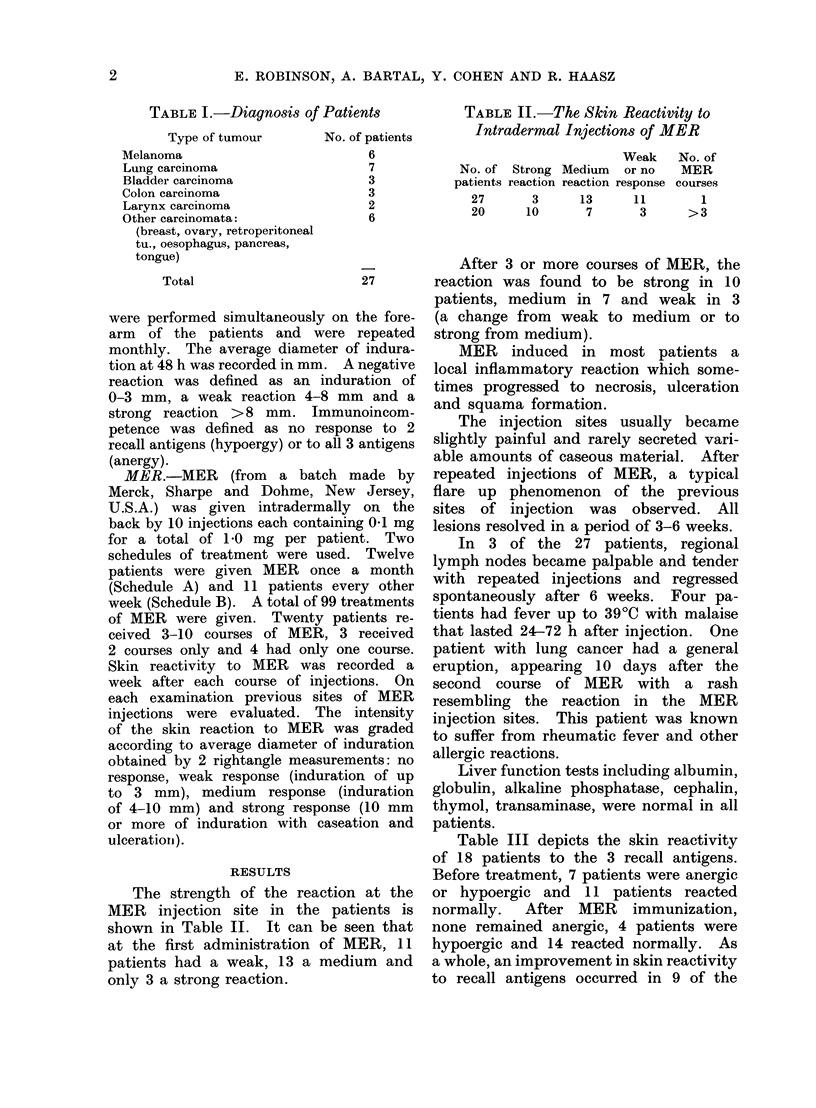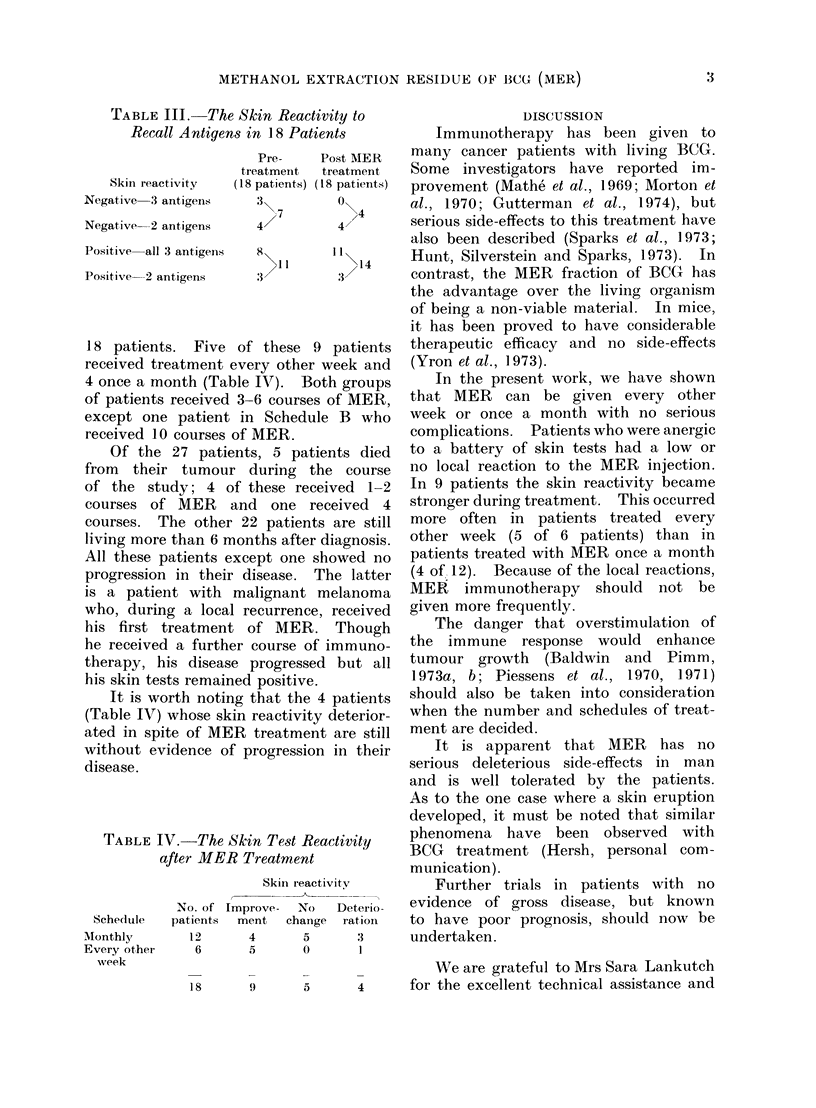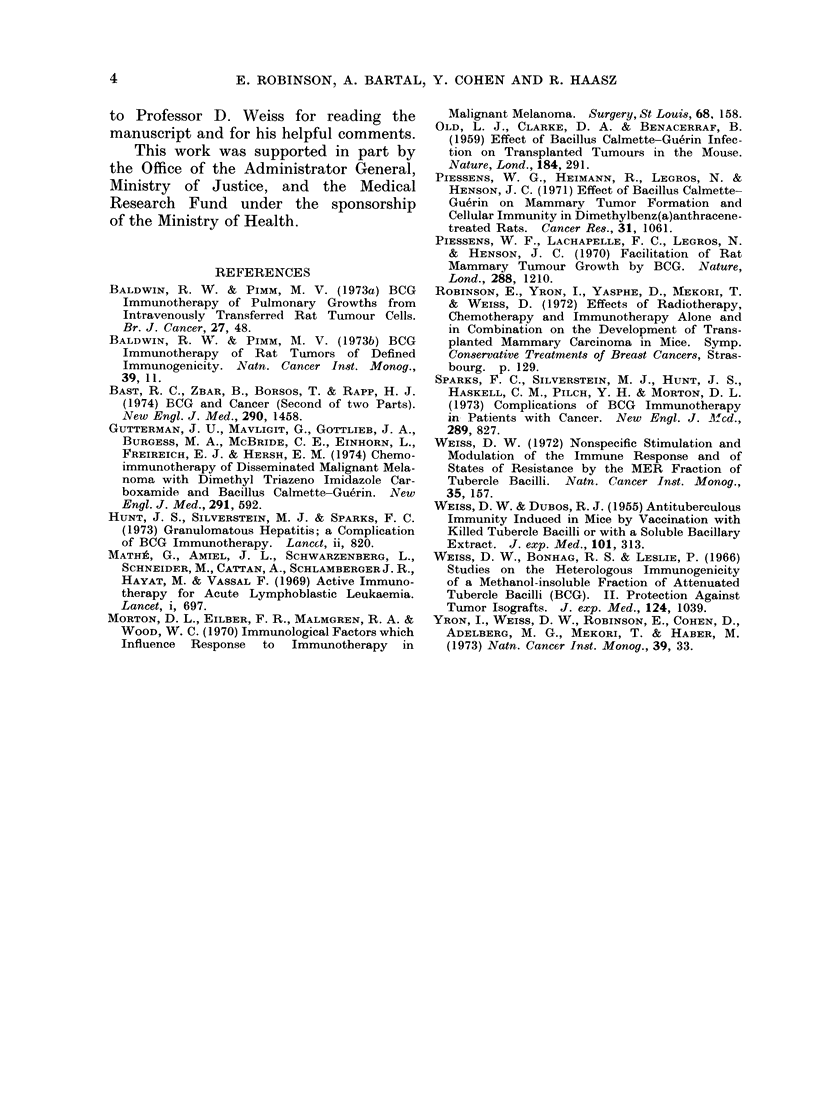# A preliminary report on the effects of methanol extraction residue of BCG (MER) on cancer patients.

**DOI:** 10.1038/bjc.1975.126

**Published:** 1975-07

**Authors:** E. Robinson, A. Bartal, Y. Cohen, R. Haasz

## Abstract

Twenty-seven patients with malignant neoplasia were injected intradermally with the methanol extraction residue (MER) fraction of tubercle bacilli. Two schedules of treatment were used: every other week and once a month; 1-10 courses of MER were administered to the patients. The skin reactivity to 3 recall antigens, as well as to the injected MER itself, was used to monitor the immune response. Improvement of skin reactivity occurred in 9 of 18 patients tested with recall antigens. Five of 6 patients treated every other week improved in their immune capacity whereas only 4 of 12 patients improved on the monthly schedule. Thus, repeated injections given every other week were more effective in increasing the cutaneous reactivity than monthly injections of MER. The side-effects of MER treatment were tolerable.


*Br. J. Cancer* (1975) **32**, 1

### A PRELIMINARY REPORT ON THE EFFECTS OF METHANOL EXTRACTION RESIDUE OF BCG (MER) ON CANCER PATIENTS

E. ROBINSON*, A. BARTAL, Y. COHEN AND R. HAASZ

*From the Department of Oncology, Rambam University Hospital and Aba Khoushy School of Medicine, The Technion, Haifa, Israel*

Received 28 January 1975. Accepted 10 March 1975

**Summary.—Twenty-seven patients with malignant neoplasia were injected intradermally with the methanol extraction residue (MER) fraction of tubercle bacilli. Two schedules of treatment were used: every other week and once a month; 1–10 courses of MER were administered to the patients. The skin reactivity to 3 recall antigens, as well as to the injected MER itself, was used to monitor the immune response. Improvement of skin reactivity occurred in 9 of 18 patients tested with recall antigens. Five of 6 patients treated every other week improved in their immune capacity whereas only 4 of 12 patients improved on the monthly schedule. Thus, repeated injections given every other week were more effective in increasing the cutaneous reactivity than monthly injections of MER. The side-effects of MER treatment were tolerable.**

THE SUCCESSFUL use of BCG (bacillus Calmette–Guérin) in the treatment of animals and humans bearing tumours has been reported (Old, Clarke and Benacerraf, 1959; Mathé *et al.*, 1969; Bast *et al.*, 1974). MER is a methanol extraction residue of BCG; it is not viable and has a broad activity as a stimulant of immunological reactivity and of resistance to microbial pathogens and neoplastic cells (Weiss and Dubos, 1955; Weiss, Bonhag and Leslie, 1966; Weiss, 1972; Robinson *et al.*, 1972).

Previously, we have reported in tumour bearing mice that radiotherapy and chemotherapy combined with MER treatment tend to be more effective in reducing tumour volume and prolonging the survival of the animals than radio- or chemotherapy alone (Yron *et al.*, 1973). In the present study of inoperable cancer patients, we have evaluated the tolerance to MER, its effect on the disease and the skin reactivity to recall antigens.

### MATERIALS AND METHODS

*Patients.*—Twenty-seven patients with histologically confirmed malignant neoplasia were included in this study; Table I shows the diagnoses. Twenty-one of the patients suffered from inoperable advanced epithelial cancer and 6 with malignant melanoma; all melanoma patients underwent resection of the primary and metastatic regional lymph nodes and had no evidence of disease. All the patients except one had a life expectancy of more than 3 months. The patients were examined at least every 2 weeks and received conventional treatment, radio- and/or chemotherapy according to standard protocols used in our department. The informed consent of the patients was obtained before admission to the study.

*Test antigens.*—Eighteen patients were skin tested by intradermal injection of 0·1 ml of 3 recall antigen preparations. The antigens were purified protein derivative (Ministry of Health, Israel, 2TU), streptokinase, streptodornase (Lederle, U.S.A., 40 $\mu$/ 10 $\mu$), and candida (Institute of Biology, Nes Ziona, Israel, 0·1%). The skin tests

* Associate Professor and Established Investigator of the Chief Scientist's Bureau, Ministry of Health.

TABLE I.—*Diagnosis of Patients*

| Type of tumour | No. of patients |
|---|---|
| Melanoma | 6 |
| Lung carcinoma | 7 |
| Bladder carcinoma | 3 |
| Colon carcinoma | 3 |
| Larynx carcinoma | 2 |
| Other carcinomata: (breast, ovary, retroperitoneal tu., oesophagus, pancreas, tongue) | 6 |
| Total | 27 |

were performed simultaneously on the forearm of the patients and were repeated monthly. The average diameter of induration at 48 h was recorded in mm. A negative reaction was defined as an induration of 0–3 mm, a weak reaction 4–8 mm and a strong reaction >8 mm. Immunoincompetence was defined as no response to 2 recall antigens (hypoergy) or to all 3 antigens (anergy).

*MER*.—MER (from a batch made by Merck, Sharpe and Dohme, New Jersey, U.S.A.) was given intradermally on the back by 10 injections each containing 0·1 mg for a total of 1·0 mg per patient. Two schedules of treatment were used. Twelve patients were given MER once a month (Schedule A) and 11 patients every other week (Schedule B). A total of 99 treatments of MER were given. Twenty patients received 3–10 courses of MER, 3 received 2 courses only and 4 had only one course. Skin reactivity to MER was recorded a week after each course of injections. On each examination previous sites of MER injections were evaluated. The intensity of the skin reaction to MER was graded according to average diameter of induration obtained by 2 rightangle measurements: no response, weak response (induration of up to 3 mm), medium response (induration of 4–10 mm) and strong response (10 mm or more of induration with caseation and ulceration).

### RESULTS

The strength of the reaction at the MER injection site in the patients is shown in Table II. It can be seen that at the first administration of MER, 11 patients had a weak, 13 a medium and only 3 a strong reaction.

TABLE II.—*The Skin Reactivity to Intradermal Injections of MER*

| No. of patients | Strong reaction | Medium reaction | Weak or no response | No. of MER courses |
|---|---|---|---|---|
| 27 | 3 | 13 | 11 | 1 |
| 20 | 10 | 7 | 3 | >3 |

After 3 or more courses of MER, the reaction was found to be strong in 10 patients, medium in 7 and weak in 3 (a change from weak to medium or to strong from medium).

MER induced in most patients a local inflammatory reaction which sometimes progressed to necrosis, ulceration and squama formation.

The injection sites usually became slightly painful and rarely secreted variable amounts of caseous material. After repeated injections of MER, a typical flare up phenomenon of the previous sites of injection was observed. All lesions resolved in a period of 3–6 weeks.

In 3 of the 27 patients, regional lymph nodes became palpable and tender with repeated injections and regressed spontaneously after 6 weeks. Four patients had fever up to 39°C with malaise that lasted 24–72 h after injection. One patient with lung cancer had a general eruption, appearing 10 days after the second course of MER with a rash resembling the reaction in the MER injection sites. This patient was known to suffer from rheumatic fever and other allergic reactions.

Liver function tests including albumin, globulin, alkaline phosphatase, cephalin, thymol, transaminase, were normal in all patients.

Table III depicts the skin reactivity of 18 patients to the 3 recall antigens. Before treatment, 7 patients were anergic or hypoergic and 11 patients reacted normally. After MER immunization, none remained anergic, 4 patients were hypoergic and 14 reacted normally. As a whole, an improvement in skin reactivity to recall antigens occurred in 9 of the

TABLE III.—*The Skin Reactivity to Recall Antigens in* 18 *Patients*

| Skin reactivity | Pre-treatment (18 patients) | Post MER treatment (18 patients) |
|---|---|---|
| Negative—3 antigens | 3 } 7 | 0 } 4 |
| Negative—2 antigens | 4 | 4 |
| Positive—all 3 antigens | 8 } 11 | 11 } 14 |
| Positive—2 antigens | 3 | 3 |

18 patients. Five of these 9 patients received treatment every other week and 4 once a month (Table IV). Both groups of patients received 3–6 courses of MER, except one patient in Schedule B who received 10 courses of MER.

Of the 27 patients, 5 patients died from their tumour during the course of the study; 4 of these received 1–2 courses of MER and one received 4 courses. The other 22 patients are still living more than 6 months after diagnosis. All these patients except one showed no progression in their disease. The latter is a patient with malignant melanoma who, during a local recurrence, received his first treatment of MER. Though he received a further course of immunotherapy, his disease progressed but all his skin tests remained positive.

It is worth noting that the 4 patients (Table IV) whose skin reactivity deteriorated in spite of MER treatment are still without evidence of progression in their disease.

TABLE IV.—*The Skin Test Reactivity after MER Treatment*

| Schedule | No. of patients | Skin reactivity: Improvement | No change | Deterioration |
|---|---|---|---|---|
| Monthly | 12 | 4 | 5 | 3 |
| Every other week | 6 | 5 | 0 | 1 |
| | 18 | 9 | 5 | 4 |

DISCUSSION

Immunotherapy has been given to many cancer patients with living BCG. Some investigators have reported improvement (Mathé *et al.*, 1969; Morton *et al.*, 1970; Gutterman *et al.*, 1974), but serious side-effects to this treatment have also been described (Sparks *et al.*, 1973; Hunt, Silverstein and Sparks, 1973). In contrast, the MER fraction of BCG has the advantage over the living organism of being a non-viable material. In mice, it has been proved to have considerable therapeutic efficacy and no side-effects (Yron *et al.*, 1973).

In the present work, we have shown that MER can be given every other week or once a month with no serious complications. Patients who were anergic to a battery of skin tests had a low or no local reaction to the MER injection. In 9 patients the skin reactivity became stronger during treatment. This occurred more often in patients treated every other week (5 of 6 patients) than in patients treated with MER once a month (4 of 12). Because of the local reactions, MER immunotherapy should not be given more frequently.

The danger that overstimulation of the immune response would enhance tumour growth (Baldwin and Pimm, 1973*a*, *b*; Piessens *et al.*, 1970, 1971) should also be taken into consideration when the number and schedules of treatment are decided.

It is apparent that MER has no serious deleterious side-effects in man and is well tolerated by the patients. As to the one case where a skin eruption developed, it must be noted that similar phenomena have been observed with BCG treatment (Hersh, personal communication).

Further trials in patients with no evidence of gross disease, but known to have poor prognosis, should now be undertaken.

We are grateful to Mrs Sara Lankutch for the excellent technical assistance and to Professor D. Weiss for reading the manuscript and for his helpful comments.

This work was supported in part by the Office of the Administrator General, Ministry of Justice, and the Medical Research Fund under the sponsorship of the Ministry of Health.

### REFERENCES

BALDWIN, R. W. & PIMM, M. V. (1973*a*) BCG Immunotherapy of Pulmonary Growths from Intravenously Transferred Rat Tumour Cells. *Br. J. Cancer*, **27**, 48.

BALDWIN, R. W. & PIMM, M. V. (1973*b*) BCG Immunotherapy of Rat Tumors of Defined Immunogenicity. *Natn. Cancer Inst. Monog.*, **39**, 11.

BAST, R. C., ZBAR, B., BORSOS, T. & RAPP, H. J. (1974) BCG and Cancer (Second of two Parts). *New Engl. J. Med.*, **290**, 1458.

GUTTERMAN, J. U., MAVLIGIT, G., GOTTLIEB, J. A., BURGESS, M. A., MCBRIDE, C. E., EINHORN, L., FREIREICH, E. J. & HERSH, E. M. (1974) Chemoimmunotherapy of Disseminated Malignant Melanoma with Dimethyl Triazeno Imidazole Carboxamide and Bacillus Calmette–Guérin. *New Engl. J. Med.*, **291**, 592.

HUNT, J. S., SILVERSTEIN, M. J. & SPARKS, F. C. (1973) Granulomatous Hepatitis; a Complication of BCG Immunotherapy. *Lancet*, ii, 820.

MATHÉ, G., AMIEL, J. L., SCHWARZENBERG, L., SCHNEIDER, M., CATTAN, A., SCHLAMBERGER J. R., HAYAT, M. & VASSAL F. (1969) Active Immunotherapy for Acute Lymphoblastic Leukaemia. *Lancet*, i, 697.

MORTON, D. L., EILBER, F. R., MALMGREN, R. A. & WOOD, W. C. (1970) Immunological Factors which Influence Response to Immunotherapy in Malignant Melanoma. *Surgery, St Louis*, **68**, 158.

OLD, L. J., CLARKE, D. A. & BENACERRAF, B. (1959) Effect of Bacillus Calmette–Guérin Infection on Transplanted Tumours in the Mouse. *Nature, Lond.*, **184**, 291.

PIESSENS, W. G., HEIMANN, R., LEGROS, N. & HENSON, J. C. (1971) Effect of Bacillus Calmette–Guérin on Mammary Tumor Formation and Cellular Immunity in Dimethylbenz(a)anthracene-treated Rats. *Cancer Res.*, **31**, 1061.

PIESSENS, W. F., LACHAPELLE, F. C., LEGROS, N. & HENSON, J. C. (1970) Facilitation of Rat Mammary Tumour Growth by BCG. *Nature, Lond.*, **288**, 1210.

ROBINSON, E., YRON, I., YASPHE, D., MEKORI, T. & WEISS, D. (1972) Effects of Radiotherapy, Chemotherapy and Immunotherapy Alone and in Combination on the Development of Transplanted Mammary Carcinoma in Mice. Symp. *Conservative Treatments of Breast Cancers*, Strasbourg. p. 129.

SPARKS, F. C., SILVERSTEIN, M. J., HUNT, J. S., HASKELL, C. M., PILCH, Y. H. & MORTON, D. L. (1973) Complications of BCG Immunotherapy in Patients with Cancer. *New Engl. J. Med.*, **289**, 827.

WEISS, D. W. (1972) Nonspecific Stimulation and Modulation of the Immune Response and of States of Resistance by the MER Fraction of Tubercle Bacilli. *Natn. Cancer Inst. Monog.*, **35**, 157.

WEISS, D. W. & DUBOS, R. J. (1955) Antituberculous Immunity Induced in Mice by Vaccination with Killed Tubercle Bacilli or with a Soluble Bacillary Extract. *J. exp. Med.*, **101**, 313.

WEISS, D. W., BONHAG, R. S. & LESLIE, P. (1966) Studies on the Heterologous Immunogenicity of a Methanol-insoluble Fraction of Attenuated Tubercle Bacilli (BCG). II. Protection Against Tumor Isografts. *J. exp. Med.*, **124**, 1039.

YRON, I., WEISS, D. W., ROBINSON, E., COHEN, D., ADELBERG, M. G., MEKORI, T. & HABER, M. (1973) *Natn. Cancer Inst. Monog.*, **39**, 33.